# Simplified Visual Stimuli Impair Retrieval and Transfer in Audiovisual Equivalence Learning Tasks

**DOI:** 10.1002/brb3.70339

**Published:** 2025-02-19

**Authors:** Kálmán Tót, Gabriella Eördegh, Noémi Harcsa‐Pintér, Balázs Bodosi, Szabolcs Kéri, Ádám Kiss, András Kelemen, Gábor Braunitzer, Attila Nagy

**Affiliations:** ^1^ Department of Physiology, Albert Szent‐Györgyi Medical School University of Szeged Szeged Hungary; ^2^ Department of Theoretical Health Sciences and Health Management, Faculty of Health Sciences and Social Studies University of Szeged Szeged Hungary; ^3^ Department of Technical Informatics University of Szeged Szeged Hungary; ^4^ Nyírő Gyula Hospital Laboratory for Perception & Cognition and Clinical Neuroscience Budapest Hungary; ^5^ Department of Cognitive Science Budapest University of Technology and Economics Budapest Hungary

**Keywords:** associative learning, audiovisual, human, psychophysics, stimulus verbalizability, visual

## Abstract

**Background:**

The visually guided Rutgers Acquired Equivalence Test (RAET) and the various visual and audiovisual versions of the test with the same structure involve rule acquisition, retrieval, and generalization and is based on learning stimulus pairs (antecedents and consequents). In an earlier study we have found no difference in the acquisition learning and only slight enhancement in retrieval and generalization in the audiovisual learning compared to the visual one if complex readily verbalizable visual stimuli (cartoon faces and color fish) were used. In this study, we sought to examine whether similar phenomena can be observed with feature‐restricted, less verbalizable visual stimuli (geometric shapes).

**Methods:**

A total of 119 healthy adult volunteers completed two computer‐based test paradigms: Polygon (PO) and SoundPolygon (SP). PO is a visual test where the antecedents are shaded circles, and the consequents are geometric shapes. SP is an audiovisual test where the antecedents are sounds and the consequents are the same geometric shapes as in PO.

**Results:**

There were no significant differences in the performances and the reaction times in the acquisition phase between the PO (visual) and SP (audiovisual) tests. However, the performances in retrieval and generalization were significantly poorer in the audiovisual test and the reaction times were also longer.

**Conclusion:**

The acquisition phase seems to be independent from the stimulus modality if the simple geometric shapes were visual stimuli. However, feature‐restricted, less verbalizable visual stimuli make more difficult to retrieve and generalize the already acquired audiovisual information.

## Graphical abstract

The study examined how simplified visual stimuli affect learning, retrieval, and generalization (transfer) in visual and audiovisual acquired equivalence tests. No differences were found in acquisition between the two tests, but retrieval and generalization were significantly poorer in the audiovisual test. These suggest that simple, less verbalizable visual stimuli make it harder to retrieve and transfer audiovisual information.

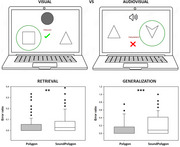

## 1 Introduction

Equivalence learning is an essential cognitive function in humans and animals. Two discrete and often different percepts (antecedents) are linked together based on a shared outcome or consequence (Molet et al. 2012; Ward‐Robinson and Hall 1999). Myers et al. have created the Rutgers Acquired Equivalence Test (RAET) to study how humans with basal ganglia or hippocampus disorders learn equivalence through visual cues (Myers et al. 2003). The test is computer‐based and has two phases: acquisition and test. In the acquisition phase, the participant matches two visual stimuli and receives feedback on their choices. This helps them learn the pairing rule. In the test phase, the participant recalls the learned associations (retrieval) and creates new ones based on predictable patterns, a process known as generalization (or transfer in animal studies; we use these two terms interchangeably here). In the literature, a similar task is referred to as the matching‐to‐sample task, where the terms “antecedent” and “consequent” correspond to what are commonly called the “sample stimulus” and “comparison stimuli,” respectively (Anderson and Colombo 2019). The acquisition phase is associated with frontal–striatal loops, while the test phase is linked primarily to the hippocampi and the medial temporal lobe (Cohen et al. 1999; Gogtay et al. 2006; Larsen and Luna 2015; Moustafa et al. 2010; Moustafa et al. 2009; Persson et al. 2014; Porter et al. 2015). These structures are also involved in multisensory processing (Bates and Wolbers 2014; Chudler et al. 1995; Nagy et al. 2006; Schwarz et al. 1984).

In our previous studies, we demonstrated the effects of stimulus modality (visual, auditory, or audiovisual) and visual stimulus complexity on equivalence learning in various versions of the RAET paradigm, all developed in our laboratory (Eördegh et al. 2019, 2022; Tót et al. 2022). In particular, we showed that visual stimuli poor in easily verbalizable stimulus features (feature‐restricted stimuli: line drawings of geometric shapes) allowed significantly poorer equivalence acquisition than stimuli rich in such features (nonrestricted stimuli: male and female cartoon faces of adults and children with different hair colors; Eördegh et al. 2022). We also demonstrated that the use of such feature‐restricted visual stimuli as consequents was associated with poorer association learning than the use of nonrestricted visual stimuli if the task was audiovisual and the antecedents were sounds (Tót et al. 2022). Finally, we showed that the multimodality of the task (i.e., when the antecedents were sounds and the consequents were nonfeature‐restricted visual stimuli) had no effect on performance in the acquisition phase of RAET, but could slightly enhance retrieval and generalization (Eördegh et al. 2019). Therefore, it is logical to ask if the observed multisensory enhancement can also be seen if feature‐restricted visual stimuli are used as consequents. In this study, we sought to answer this question by comparing volunteers’ performance in a purely visual and an audiovisual version of RAET.

## 2 Methods

### 2.1 Subjects

Altogether 119 healthy adult volunteers participated in the study (50 females and 69 males, mean age: 28 ± 13.02 years, range: 18–68 years; six participants were over the age of 60). The required sample size was calculated in G*Power 3.1.9.2 (RRID:SCR_013726). The minimum required sample size was 47, assuming *p* < 0.05, 1 − *β* = 0.95, and an effect size of 0.5.

However, we collected data not only for the comparison presented in this study but also for a crossover analysis between these and earlier tests with more complex visual stimuli. As a result, we had a larger sample size than the calculated minimum for the current comparison. We argue that the results are clearer and more reliable with a sample size larger than the minimum required.

The participants were recruited on a voluntary basis. The volunteers received no compensation (monetary or otherwise) and were free to quit at any time. The participants were informed about the aims and procedures of the study and gave their written informed consent. Only volunteers free of any psychiatric, neurological, otologic, or ophthalmologic disorder (either at the time of the recruitment or in history) were eligible. Before testing, samples of the visual and auditory stimuli were shown to the participants, to confirm that they could see and hear them properly. The study conformed to the Declaration of Helsinki in all respects and was approved by the Regional Research Ethics Committee for Medical Research at the University of Szeged, Hungary (27/2020‐SZTE).

### 2.2 The Paradigm and the Applied Tests

The tests were administered to the participants on a laptop (Lenovo ThinkBook 15‐IIL, Lenovo, China). In the case of the audiovisual test, overear headphones were used (Sennheiser HD439, Sennheiser Germany). The participants were tested alone in a quiet room, sitting at a comfortable distance (57 cm) from the screen. No forced quick responses were expected from them to avoid performance anxiety. The participants completed both tests one after another without any rest period, in alternating order.

Both tests are variants of the RAET, a visual equivalence learning test paradigm developed by Myers et al. (2003). Our group has worked with this paradigm for more than a decade now. In this period, after working with the original test for some time, we have prepared a Hungarian version in Assembly for Windows, developed an audiovisual version, and started to manipulate the complexity/verbalizability of the visual stimuli to see if such manipulations influence test performance (Eördegh et al. 2019, 2022; Tót et al. 2022; Öze et al. 2017). These modifications were made with the permission of the authors.

Regardless of the stimuli used, the paradigm consists of two main phases: the acquisition phase and the test phase. The acquisition phase has three stages: shaping, equivalence learning, and the introduction of new consequents, while the test phase has two stages: retrieval and transfer.

**FIGURE 1:**
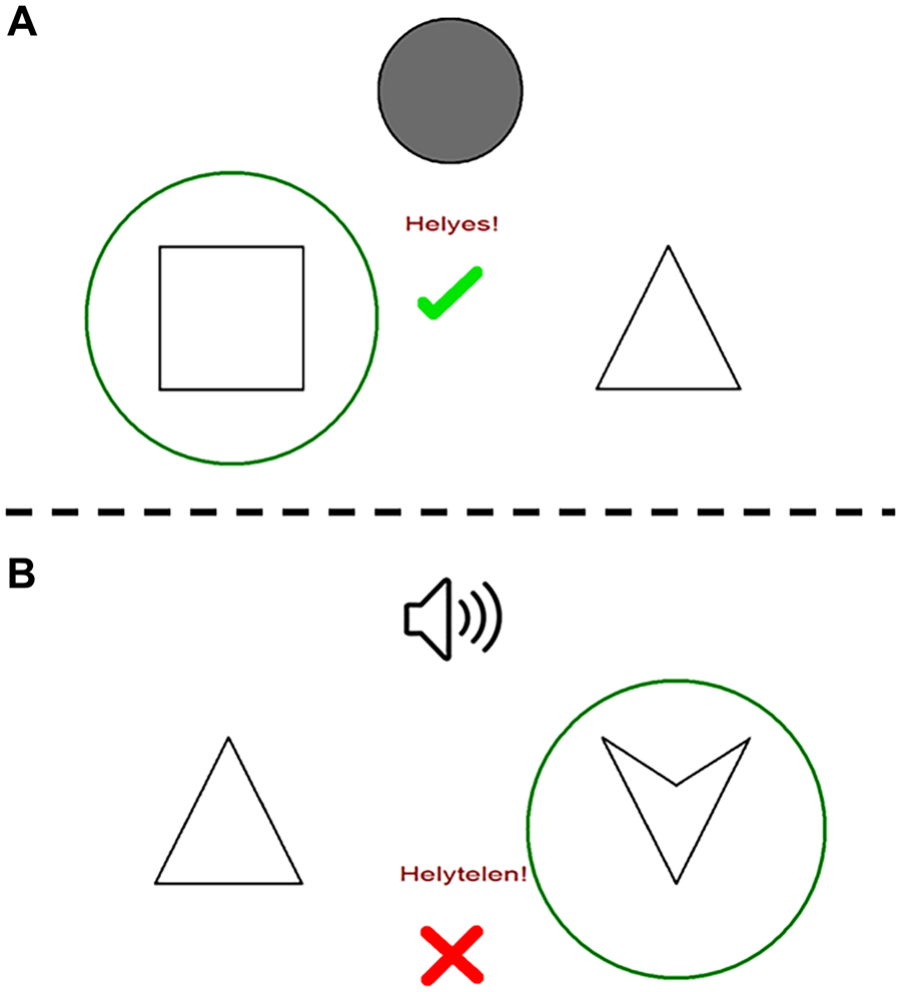
A trial in the acquisition phase of Polygon (A) and SoundPolygon (B).

In the acquisition phase, the participants learn the associations between antecedent and consequent stimuli through trial and error. In this phase, the program gives feedback about the correctness of the responses. When a response is correct, a green check mark appears with the word “helyes!” (Correct!) written under it, and when a response is incorrect, a red X with the word “helytelen!” (“Incorrect!”) written under it is shown (Figure 1).

In each trial, the subject simultaneously sees or hears the antecedent stimulus (circles or sounds, respectively), and sees two consequents (geometric shapes) on the left and right side of the screen. Then the subject gives a response which consequent belongs to the given antecedent by pressing the “left” or “right” button. The choice is indicated with the green circle. Immediate visual feedback is given. A green checkmark with the word Helyes! (Correct!) indicates a correct guess and a red X with the word Helytelen! (Incorrect!) indicates an incorrect guess.

The first stage of the acquisition phase is shaping. In this part, subjects learn single stimulus pairs and the fact that there is a pairing rule. For instance, when antecedent A1 is presented, the consequent is X1, and when antecedent B1 is presented, the consequent is Y1. Shaping is followed by the second stage, equivalence training. Two new antecedents (A2 and B2) are introduced, and the subject learns that A2, like A1, is paired with X1, and that B2, like B1, is paired with Y1. This way, the equivalence between the antecedents (A1; A2) and (B1; B2) is established. Finally, in the third stage, a new pair of consequents (X2 and Y2) are introduced. The subject learns to memorize that X2 is paired with A1, and Y2 is paired with B1. This way A1 and B1 gain an additional consequent. At this point, the subject knows that antecedent A1 is paired with consequents X1 and X2 and antecedent B1 is paired with consequents Y1 and Y2, and this is where the acquisition phase ends. Throughout the acquisition phase, after introducing a new association, the participant must give a certain number of subsequent correct answers (4, 6, 8, 10, and 12 after each new association, respectively) to proceed. Because of this, the number of trials in this phase is not constant, and it is an indicator of the participant's learning performance.

There are altogether eight possible antecedent–consequent pairs, of which six are presented in the acquisition phase; the remaining two pairs appear in the transfer stage of the test phase (see below).

When the participant completes the acquisition phase, a page will appear with a statement indicating that the learning phase is over and that no further feedback will be given in the following section. Once the participant has read this, they can proceed to the test phase.

In the test phase, no feedback is given about the correctness of the responses anymore. In this phase, the participant must recall the already learned stimulus pairs (retrieval), and the remaining two pairs (A2:X2 and B2:Y2) are presented (transfer or generalization). The participant is not informed about the new stimulus pairs. In the case of successful acquisition, the participant will correctly guess that X2 is the right choice when A2 is presented, and Y2 when B2 is presented, even though these pairs have not been presented before. For the purposes of analysis, retrieval and generalization are treated as separate stages of the test phase. However, in fact, the familiar and unfamiliar pairs are presented together, in random order. There are 48 trials in the test phase, of which 36 are retrieval and 12 are generalization trials. In contrast to the acquisition phase, these numbers are fixed for all participants.

This study used two specific versions of the paradigm: Polygon (PO) and SoundPolygon (SP). Both versions have been developed in our laboratory.

PO is a purely visual test: both the antecedents and the consequents are visual stimuli. The antecedents are circles filled with different shades: white (e.g., A1), light gray (e.g., A2), dark gray (e.g., B1), and black (e.g., B2). The consequents are different geometric shapes with no color information: a triangle (e.g., X1), a square (e.g., X2), a rhombus (e.g., Y1), and a concave deltoid (e.g., Y2).

In each trial, an antecedent stimulus appears in the middle of the screen, and two possible consequents are shown on the left and right sides below it. The participant must guess which geometric shape belongs to the presented circle by pressing either the “left” or the “right” button (corresponding to the side on which the given consequent stimulus is shown). The stimuli remain on the screen until the participant has made a choice (Figure 1).

SP is an audiovisual test: the antecedents are sounds and the consequents are visual stimuli. The antecedents are a cat's meow (e.g., A1), a guitar chord (e.g., A2), the sound of a vehicle (e.g., B1), and a female voice saying “Hey” (e.g., B2). Before the testing session, the stimuli were shown to the participants to make sure that they could hear them correctly.

The consequents are the same geometric shapes as in PO. In each trial, the participant simultaneously hears a sound and sees two geometric shapes on the left and right sides of the screen. The participant must guess which geometric shape belongs to the presented circle by pressing either the “left” or the “right” button (corresponding to the side on which the given consequent stimulus is shown). The duration of the auditory antecedent is 1500 ms, and the visual consequents remain on the screen until the participant has made a choice.

**FIGURE 2:**
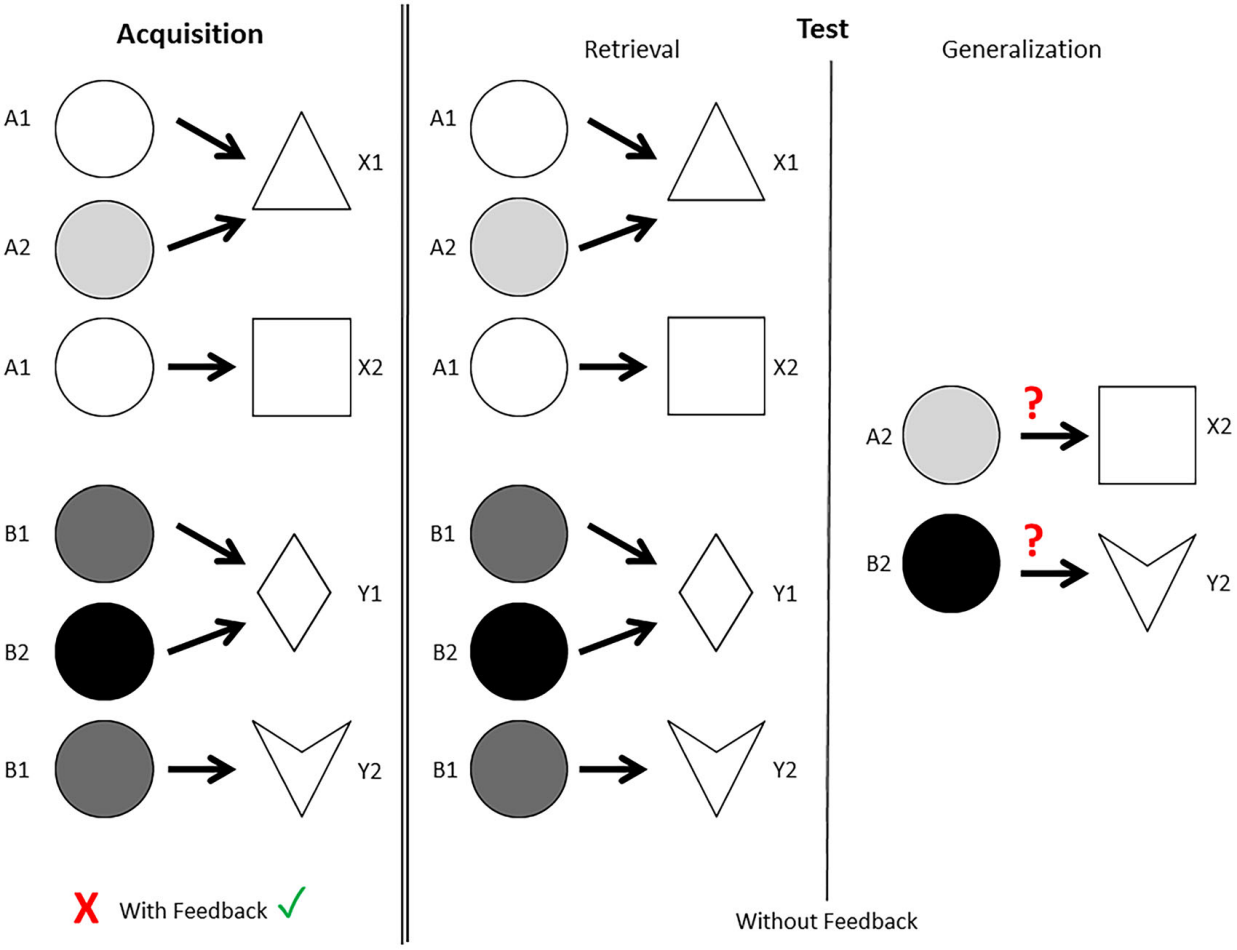
Overview structure of the Polygon test. The antecedents are grayscale circles: white (A1), light gray (A2), dark gray (B1), and black (B2). The consequents are simple geometric shapes: a triangle (X1), a square (X2), a rhombus (Y1), and a concave deltoid (Y2). The test has two phases: the acquisition phase (with feedback) and the test phase (without any further feedback). In the later participants have to retrieve the learned associations and generalize the equivalence rule to new stimulus pairs.

**FIGURE 3:**
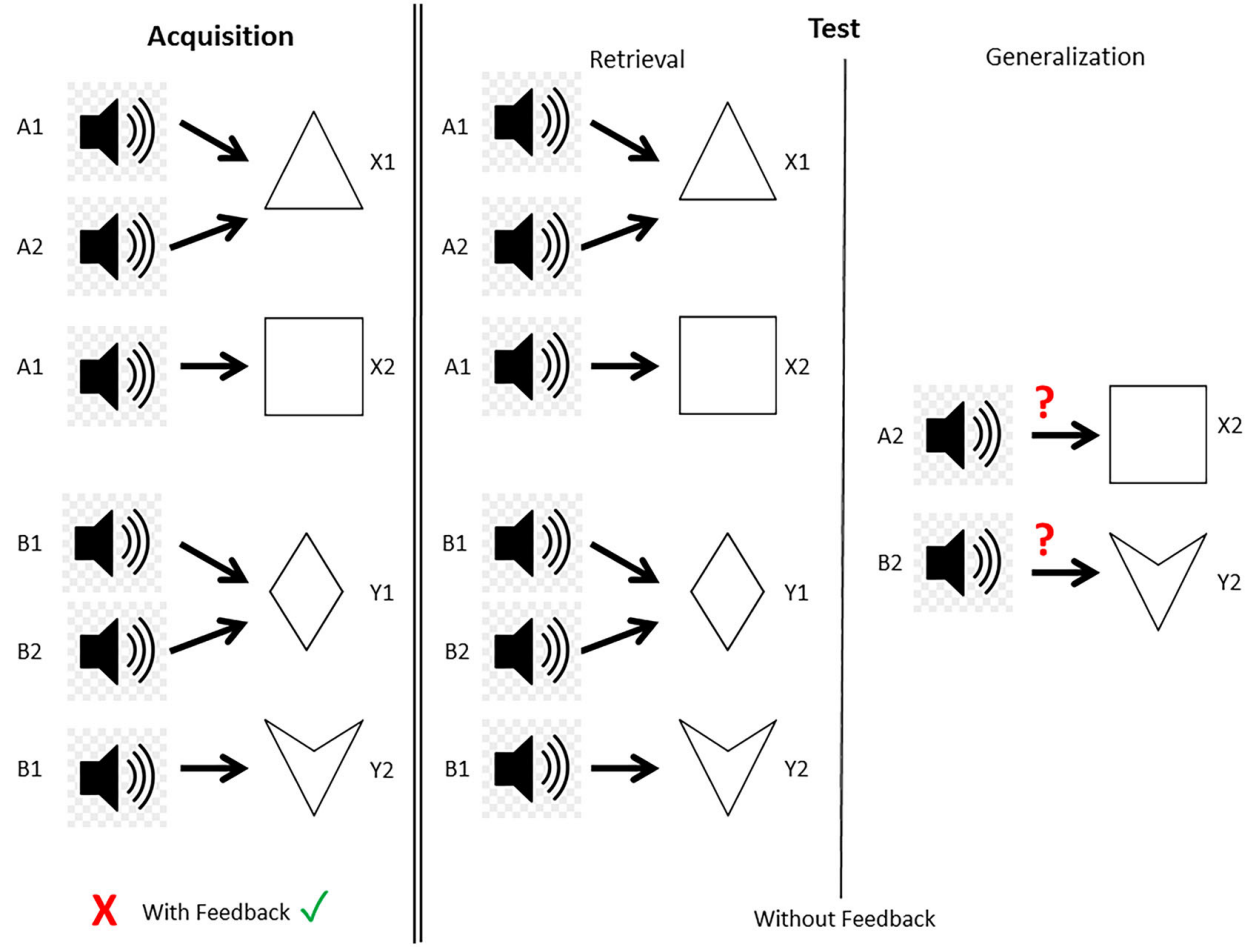
Overview structure of the SoundPolygon test. The antecedent stimuli are sounds of a cat (A1), a guitar chord (A2), the sound of a vehicle (B1), and a woman's voice (B2). The consequents are the same as in the Polygon test, and the structure is also consistent with it.

The tests are summarized in Figures 2 and 3.

### 2.3 Data Analysis

The performance of the participants was characterized by four main parameters: the number of trials necessary for the completion of the acquisition phase (NAT), association learning error rate (the ratio of the incorrect choices during the acquisition trials, ALER), retrieval error rate (RER), and generalization error rate (GER). Error rates were calculated by dividing the number of incorrect responses by the total number of trials. Reaction times were recorded with millisecond accuracy for each trial, and they were analyzed for the acquisition, retrieval, and generalization trials separately. Reaction time (RT) was defined as the time that elapsed between the appearance of the stimuli and the participant's response. Only RTs of the correct choices were included, and values over 3SD were excluded. Statistical analysis was performed in Statistica 13.4.0.14 (TIBCO Software Inc., USA). All the parameters mentioned above were compared between the two paradigms. As the data were non‐normally distributed (Shapiro–Wilk *p* < 0.05), the Wilcoxon matched‐pairs test was used for the hypothesis tests. Effect sizes (*d*) and post hoc power calculations were computed for significant differences using G*Power 3.1.9.7 (Universität Düsseldorf, Germany).

## 3 Results

Altogether 119 volunteers completed both tests. The analysis of their performance data is presented according to the two main phases of the test paradigm.

### 3.1 Acquisition Phase

The median number of trials required to learn the associations (NAT) in PO was 58 (range: 41–142), and in SP it was 56 (range: 41–141, Figure 4). There was no significant difference between the two tests (*Z* = 1.597, *p* = 0.110).

**FIGURE 4:**
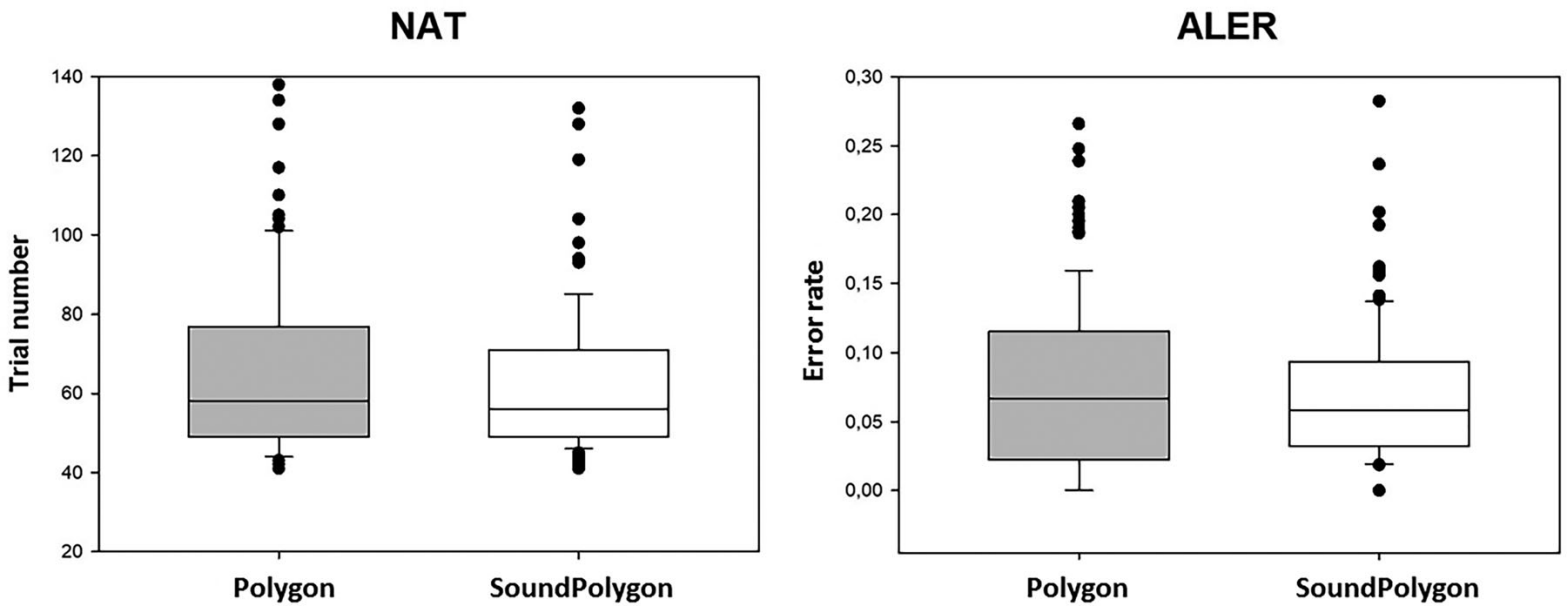
Performance in the acquisition phase in the two tests. ALER, acquisition learning error rates; NAT, the number of trials in the acquisition phase. The upper and lower margin of the boxes indicates the upper and lower quartile, respectively. The line within the boxes marks the median. The upper whiskers indicate the 90th percentile, and the lower the 10th percentiles. The dots over and under the whiskers represent the outliers.

The median of ALER (acquisition error rate) in PO was 0.067 (range: 0.00–0.27, Figure 4), and in SP it was 0.058 (range: 0.00–0.26). The difference was not significant (*Z* = 1.222, *p* = 0.222).

As for the reaction times, the median RT in PO was 1733.776 ms (range: 954.548–6330.16), and in SP it was 1783.125 ms (range: 1056.783–4762.33). The difference was not significant in this parameter either (*Z* = 1.352, *p* = 0.176).

### 3.2 Test Phase

**FIGURE 5:**
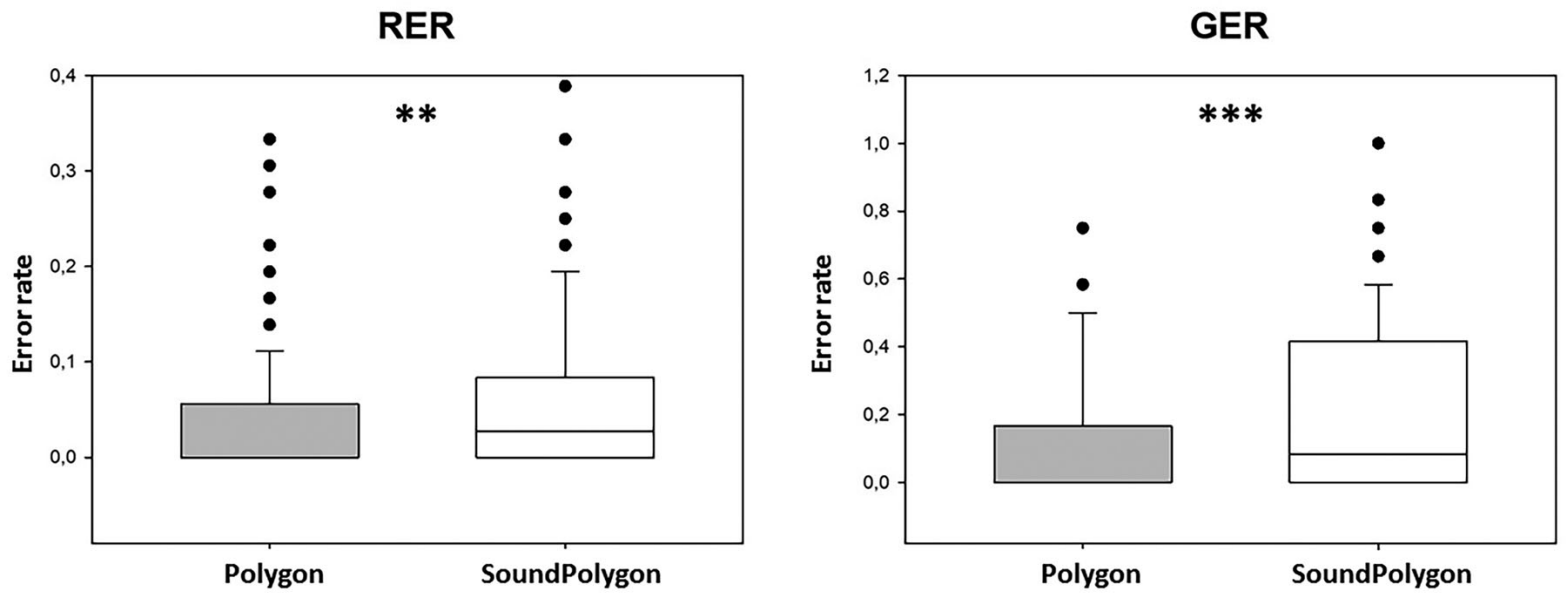
Performance in the test phase in the two tests. GER, generalization error rate; RER, retrieval error rate. Asterisks indicate the significant difference: ** at the level *p* < 0.01, and *** at the level of *p* < 0.001. The other conventions are the same as in Figure 1.

Contrary to the acquisition phase, significant difference was found between PO and SP in both RER (retrieval error rate) and GER (generalization error rate). The median of RER (Figure 5) was 0.00 (range: 0.00–0.33) in PO, and 0.028 (range: 0.00–0.39) in SP (*Z* = 2.599, *p* = 0.009, Power: 0.726, effect size: 0.2988).

In PO, the median of GER (Figure 5) was 0.00 (range: 0.00–0.75), and 0.083 (range: 0.00–1.00) in SP (*Z* = 3.630, *p* = 0.0003, Power: 0.9335, effect size: 0.4188).

The reaction times also differed significantly between the two tests. The median RT for retrieval in PO was 1794.806 ms (range: 902.417–4293.31), 1836.056 ms (range: 1028.611–5298.78) in SP (*Z* = 2.079, *p* = 0.038, Power: 0.3938, effect size: 0.1830). The median RT for generalization in PO was 2294.250 ms (range: 902.500–7434.92) and 2514.250 ms (range: 1071.750–18381.00) in SP (*Z* = 2.595, *p* = 0.009, Power: 0.6623, effect size: 0.2746).

## 4 Discussion

The idea of this study arose from a previous study of our research group in connection with the RAET paradigm. In that study, we observed that the multimodality of the task (i.e., when the antecedents were sounds and the consequents were nonfeature‐restricted visual stimuli) had no effect on performance in the acquisition phase. However, it could slightly enhance retrieval and generalization (Eördegh et al. 2019). This prompted us to design a study to investigate if a similar pattern of enhancement can be observed if feature‐restricted stimuli are used as consequents. Similar to our earlier study, we found no effect on the acquisition phase. However, the findings related to the test phase (retrieval and transfer) are vastly different: the multisensory condition was associated with significant performance deterioration in this phase.

The original version of RAET (Myers et al. 2003) was created to investigate how neurological patients with basal ganglia and hippocampus dysfunction learn through visual associations. While both the basal ganglia and hippocampi process not only visual but also multisensory information (Bates and Wolbers 2014; Nagy et al. 2006, 2005; Ravassard et al. 2013), little research has been done on how multisensory information affects acquired equivalence learning.

The applied equivalence learning tasks, regardless of whether they are unimodal or multimodal, can be divided into two phases. In the acquisition phase, the subjects must learn visual and audiovisual (multisensory) antecedent–consequent stimulus pairs based on feedback. This process is associated with the frontal–striatal loops of the basal ganglia in the literature (Eördegh et al. 2019; Giricz et al. 2021; Myers et al. 2003; Packard and Knowlton 2002; Tót et al. 2022; White 1997; Öze et al. 2017). The test phase, which involves retrieval and transfer, is thought to be linked to the hippocampi and the medial temporal lobes (Myers et al. 2003; Opitz 2014).

In a previous study of our group applying nonfeature‐restricted visual stimuli (Eördegh et al. 2019), equivalence learning (the acquisition phase of RAET) was not influenced by whether the task was visual or audiovisual. This study brought the same results. No significant difference was found between the visual and audiovisual tests in any of the studied parameters, including reaction time. This finding is not self‐explanatory or obvious because it is generally held that multisensory information means more than simply the sum of the information content carried by the individual modalities (Nagy et al. 2006; van Atteveldt et al. 2014). Multimodal interaction could contribute to optimal performance in different cognitive tasks, such as visual perception (Frassinetti et al. 2002), face, voice, and object recognition (Fort et al. 2002; Love et al. 2011; Suied et al. 2009), and emotional reactions (Chen et al. 2016). Furthermore, multisensory information processing has been shown to influence reaction times, the accuracy of answering, and perceived thresholds as well (Hershenson 1962; Miller 1982; Patching and Quinlan 2004; Regenbogen et al. 2016). Nevertheless, in our study, associative equivalence learning was fairly independent of whether the applied test was visual or audiovisual. At the same time, visual consequent stimulus complexity influenced equivalence learning in both the visual and the audiovisual versions of the RAET paradigm: nonfeature‐restricted visual consequents were associated with superior performance in the acquisition phase as compared to the condition when the visual consequents were the feature‐restricted geometric shapes (Eördegh et al. 2019; Tót et al. 2022). It seems, thus, that in this paradigm, the richness of the visual consequents in readily verbalizable features is a more important determiner of acquisition than whether the task is unimodal or bimodal.

The second part of RAET is the test phase, where the participant recalls the learned associations (retrieval) and creates new ones based on predictable patterns (generalization or transfer). The retrieval part depends on the hippocampus–medial temporal lobe system (Myers et al. 2003; Opitz 2014), while the generalization part requires the hippocampi and the basal ganglia (Shohamy and Wagner 2008). Earlier, using nonfeature‐restricted visual consequents, we found that the bimodality of the test (with sounds as antecedents and cartoon faces as consequents) enhanced our subjects’ performance in the test phase as compared to the test version where both the antecedents and the consequents were visual (Eördegh et al. 2019). The most noteworthy finding of the present study is that the same is not true when feature‐restricted visual consequents are used. In fact, bimodality was associated with significantly poorer performance in the test phase as indicated by significantly increased RER and GER, as well as significantly longer reaction times. This is somewhat surprising but given that this has been observed in a relatively large sample and with proper statistical methods, we have no reason to doubt the validity of the observation.

At this point, we have too little data to offer a strong explanation for this effect, yet hypotheses may be formulated. Obviously, the degree of feature verbalizability is clearly different between the consequent stimuli used in our earlier study (Eördegh et al. 2019) and this one. We argue that the description of geometric shapes requires a much more abstract vocabulary than that of cartoon faces. As for the antecedent stimuli, these were sounds that were easy to verbalize (or at least tag verbally like: cat, guitar, car, woman). In our earlier study (Eördegh et al. 2019) when both the antecedents and consequents were easy to verbalize, it has a mild boosting effect on retrieval and generalization. However, as soon as one of them is difficult to verbalize (in the present study the geometric forms), performance in the test phase of the audiovisual test dips significantly. According to the original paper of Ashby et al. (1998) and more recent studies (Ashby and Ell 2001; Smith et al. 2012; Wasserman et al. 2023), categories may be learned in both humans and animals in a verbal (explicit) or nonverbal (implicit) way. These studies make it clear that the difficulty of the verbalizability of the rule of categorization is intimately related to the verbalizability of stimulus features: verbalizable rules are formed easily if the stimuli are easy to describe verbally. It does not seem far‐fetched to argue that it is because stimuli that are difficult to verbalize are processed predominantly in an implicit way. Hence, they are not easy to integrate into verbal rules—unlike stimuli that are easy to verbalize. Furthermore, we consider it is safe to assume that the same principle can be applied to rule learning in RAET, especially that the acquisition phase of RAET may well be regarded as the formation of categories through learning which stimuli belong together. Both our previous (Eördegh et al. 2019) and present results suggest that the conflict between the verbalizability of the antecedents and consequents does not generate any kind of difficulty in the acquisition of visual or audiovisual stimuli. On the other hand, in the present study, the decreased verbalizability of the visual consequents in the audiovisual test significantly hindered retrieval, and, in turn, operations with the information that should have been retrieved (in this case generalization, the formation of a new stimulus pair based on previous information). This is a very specific pattern, and it may follow from the structure of RAET: in the acquisition phase, the subjects receive continuous feedback on the correctness of the guesses they make, so they are not forced to make a conscious effort to acquire the rule—this phase leaves room for implicit learning. However, in the test phase, no feedback is given anymore, so the subject is forced to make a conscious effort to remember. The fact that the reaction times of the generalization part of the test phase are the longest of all parts of the test, regardless of whether the paradigm is visually or audiovisually administered, supports this (Eördegh et al. 2019). Naturally, these are only initial hypotheses regarding our observations, and the background of the observed phenomenon remains to be elucidated.

A limitation of this study is that we investigated only two sensory modalities (visual and auditory) in equivalence learning. Our aim was to compare the visual and audiovisual results using reduced visual stimuli, similar to our previous study with complex visual stimuli (Eördegh et al. 2019). However, we cannot rule out that the use of other sensory modalities could elicit different performances, which should be addressed in future studies. Another limitation of the study is that the auditory stimuli were presented for a fixed duration of 1.5 s, whereas the visual stimuli remained visible until the participant made a decision in each trial. Although this discrepancy did not affect the acquisition phase, we cannot exclude the possibility that this difference between the visual and audiovisual tests may influence reaction times in the test phase. Since reaction times are around 2 s, this could only slightly affect performance in the two tests.

Another limitation is that our conclusion is based on two comparisons: one from the current study and one from an earlier comparison. However, it would be crucial to conduct a crossover analysis among the four tests. We are currently collecting data to ensure an adequate sample size for this.

We also want to note that in this study, we aimed to investigate whether simplifying the visual stimuli affects performance. As the next step, we plan to simplify the auditory stimuli as well. This approach will allow us to differentiate between the effects of auditory and visual stimuli and determine if one has a greater impact than the other. Our intention is to make only one change at a time, as this will make it easier to draw clear conclusions from the results.

## 5 Conclusion

Our results showed that, there is no significant differences between the visual and audiovisual tests in the acquisition phase, when feature‐restricted visual consequents were applied. In contrast to the acquisition phase, the multisensory condition was associated with significant performance deterioration in both retrieval and generalization.

## Author Contributions

**Kálmán Tót**: data curation, writing–original draft, writing–review and editing, visualization, formal analysis, investigation. **Gabriella Eördegh**: conceptualization, investigation. **Noémi Harcsa‐Pintér**: investigation. **Balázs Bodosi**: investigation, software. **Szabolcs Kéri**: conceptualization. **Ádám Kiss**: formal analysis. **András Kelemen**: formal analysis. **Gábor Braunitzer**: conceptualization, writing–original draft, writing–review and editing. **Attila Nagy**: conceptualization, supervision, funding acquisition, writing–original draft, writing–review and editing.

## Conflicts of Interest

The authors declare no conflicts of interest.

### Peer Review

The peer review history for this article is available at https://publons.com/publon/10.1002/brb3.70339

## Data Availability

The data that support the findings of this study are available from the corresponding author upon reasonable request. The datasets generated during and/or analyzed during the current study are available from the corresponding author on reasonable request.
